# Comparison of two porcine acute lung injury models: a post-hoc analysis

**DOI:** 10.1186/s40635-022-00466-3

**Published:** 2022-09-05

**Authors:** René Rissel, Miriam Renz, Katja Mohnke, Julian Riedel, Katharina Ritter, Alexander Ziebart, Robert Ruemmler, Erik K. Hartmann, Jens Kamuf

**Affiliations:** Department of Anaesthesiology, Medical Centre of the Johannes Gutenberg-University, Langenbeckstraße 1, 55131 Mainz, Germany

**Keywords:** ARDS, Animal model, Acute lung injury, Pigs

## Abstract

**Background:**

Acute respiratory distress syndrome (ARDS) is a common disease in intensive care medicine. Despite intensive research, mortality rates are high, not even in COVID-19 ARDS. Thereby, pigs offer some advantages to study the characteristics of ARDS. Many different ARDS models exist. Most of the articles published focused on histopathological and microscopic lung alterations to identify the most suitable animal ARDS model. “Macroscopic” observations and descriptions are often missing. Therefore, we performed a post-hoc comparison of two common ARDS models for pigs: lipopolysaccharide (LPS) vs. a double-hit model (bronchoalveolar lavage + oleic acid infusion). We investigated hemodynamic, spirometric and laboratory changes as another main clinical part of ARDS.

**Results:**

The groups were compared by two-way analysis of variance (ANOVA) with a post-hoc Student–Newman–Keuls test. A *p* value lower than 0.05 was accepted as significant. All animals (*n* = 8 double-hit ARDS; *n* = 8 LPS ARDS) survived the observation period of 8 h. ARDS induction with reduced oxygen indices was successful performed in both models (76 ± 35/225 ± 54/212 ± 79 vs. 367 ± 64; T0/T4/T8 vs. BLH for double-hit; 238 ± 57/144 ± 59 vs. 509 ± 41; T4/T8 vs. BLH for LPS; *p* < 0.05). ARDS induced with LPS leads to more hemodynamic (mean arterial pulmonary pressure 35 ± 3/30 ± 3 vs. 28 ± 4/23 ± 4; T4/T8 LPS vs. double-hit; *p* < 0.05; doses of norepinephrine 1.18 ± 1.05 vs. 0.11 ± 0.16; LPS vs. double-hit for T8; *p* < 0.05) and inflammatory (pulmonary IL-6 expression: 2.41e−04 ± 1.08e−04 vs. 1.45e−05 ± 7.26e−06; LPS vs. double-hit; *p* < 0.05) alterations. ARDS induced by double-hit requires a more invasive ventilator strategy to maintain a sufficient oxygenation (PEEP at T4: 8 ± 3 vs. 6 ± 2; double-hit vs. LPS; *p* < 0.05).

**Conclusions:**

Both animal ARDS models are feasible and are similar to human presentation of ARDS. If your respiratory research focus on hemodynamic/inflammation variables, the LPS-induced ARDS is a feasible model. Studying different ventilator strategies, the double-hit ARDS model offers a suitable approach.

## Background

Acute respiratory distress syndrome (ARDS) are intensive care medicine syndromes characterized by pulmonary oedema, acute inflammation and haemodynamic alterations [1]. The COVID-19 pandemic has caused an increase in ARDS patients and highlighted challenges associated with this syndrome, including the lack of effective pharmacotherapy and optimal ventilator strategies [2]. Therefore, mortality for ARDS remains unacceptably high with reported rates up to 43% [2]. In respiratory research, many different animal models exist to describe the common alterations seen in human ARDS. Mice, guinea pigs and pigs are often used and show specific advantages and disadvantages [3]. Due to the lack of information to what exactly constitutes ARDS in an animal model, it is difficult for researchers to determine if they have achieved ARDS in an experimental ARDS preclinical model [4]. As a consequence, numerous promising pharmacological therapies failed to demonstrate reliable improvement in clinical outcomes, despite beneficial effects in preclinical studies [5]. In the past, only single parameters were examined in preliminary studies, for example the influence of the ARDS model on the results of the electrical impedance tomography (EIT) or the impact of pulmonary artery hypertension on right ventricular dysfunction in ARDS [6, 7]. For this reason, we compared the major “macroscopic” parameters of two animal models to specify their advantages and disadvantages in ARDS research.

## Materials and methods

Following approval of the state and institutional animal care committee (Landesuntersuchungsamt Rheinland-Pfalz, Koblenz, Germany; G16-1-015, G18-1-044, G20-1–135) eight male pigs (*Sus scrofa domesticus;* weight 30 ± 2 kg, aged 8 to 12 weeks) were prospectively examined in the vehicle group in the past 2 years for one of the two used animal models and post-hoc compared in this study (G16-1-015 = 8 vehicle animals, ARDS induced with LPS; G18-1-044/G20-1-135 = 4 vehicle animals from each study, ARDS induced with the double-hit model). The results from one of the previous mentioned studies (G18-1-044) have already been published [8]. All the cited studies were prospectively performed in compliance with the ARRIVE guidelines [9].

Anaesthesia, instrumentation and ventilator settings were conducted and performed in one lab and from one group as numerously described before and were in accordance with valid ARDS guidelines [10, 11]. The whole study and post-mortem analyses (a.e. cytokine expressions) were done investigator-blinded. At the end of the experiment, the exsanguinated left lung was weighted, sliced and dried for determination of the wet-to-dry ratio.

Starting with the ARDS induction, all animals were ventilated in a constant mode that aims to protect the lungs: tidal volume 5–7 ml kg^−1^, positive end-expiratory pressure (PEEP) 5 cmH2O and respiratory frequency 25–35 min^−1^ targeted to an end-tidal carbon dioxide level < 60 mmHg. Only the FiO_2_ was maintained at 1.0 for this phase. Afterward, FiO_2_ and PEEP were adjusted to achieve an arterial oxygen pressure (PaO_2_) ≥ 60 mmHg based on the ARDS low-PEEP table [12].

### ARDS induction with lipopolysaccharide (LPS)

After instrumentation and stabilisation of the animals baseline parameters were collected. Afterward, the ARDS was induced with the intravenous injection of 150 µg/kg/h *Escherichia coli* (*E. coli*) LPS, followed by a continuous infusion rate of 15 µg/kg/h for 8 h [13].

### ARDS induction with the double-hit

After instrumentation and stabilisation of the animals baseline parameters were assessed. For the first hit, repeated bronchoalveolar lavage (BAL) over the endotracheal tube with a 30 ml kg^−1^ of sterile balanced and heated (40° Celsius) isotonic solution (Sterofundin; B. Braun Melsungen AG, Germany) was performed. Therefore, the endotracheal tube was clamped in inspiration, the lavage set connected and immediately instilled and drained by gravity after 30 s. Fluids remaining in the endotracheal tube was suctioned afterward. The BAL procedures were repeated until a ratio of arterial partial pressure of oxygen (PaO_2_) and inspired fraction of oxygen (FiO_2_) ≤ 250 mmHg was achieved. Second, oleic acid (Ölsäure, Applichem GmbH Darmstadt, Germany) was solved in balanced electrolyte solution in a ratio of 1:10 and was then applied in fractions of 1–2 ml over 30 min. Short-term hemodynamic instability was treated by norepinephrine boli. The procedure was continued until the quotient of arterial PaO_2_ and FiO_2_ was < 100 mmHg over 15 min or until a dose maximum of 0.3 ml kg^−1^ was administered [10].

In both models ‘BLH’ represents the healthy baseline values after instrumentation, whereas ‘T0’ reflects the time of measurement directly after ARDS induction. At T0 the animals where ventilated with FiO_2_ of 1.0 and PEEP 5 to check the sustainable success of the ARDS induction. ‘T4’ and ‘T8’ represent the time four, respectively, 8 h after ARDS induction.

### Extended hemodynamic/ventilator monitoring

Hemodynamic and ventilator data collection were maintained over 8 h and saved electronically every hour (Datex S/5, GE Healthcare, Germany; Engström Carestation, GE Heathcare, USA). The hemodynamic monitoring was established in ultrasound-guided seldinger’s technique: a pulmonary artery catheter (Swan Ganz 7,5 Fr, 110 cm, Edwards Lifesciences LLC, USA) an arterial line for blood pressure monitoring and repetitive blood gas analysis, a central venous line and a pulse contour cardiac output (CO) catheter (PiCCO, Pulsion Medical, Munich, Germany) were placed via the femoral vessels. To determine the CO, a cold indicator injectate (20 ml of isotonic saline solution, 20 °C) is injected through the central venous catheter. The occurring change in the blood temperature is detected by the tip of the PiCCO catheter in the artery and the curve of the temperature change is displayed as CO on the monitor. The pulmonary artery catheter was used to measure pulmonary capillary wedge pressure and mean arterial pulmonary pressure. Development of pulmonary edema was assessed by the transpulmonary thermodilution-derived extravascular lung water index (EVLWI [ml kg^−1^]; PiCCO, Pulsion Medical, Munich, Germany). Functional residual capacity was determined semi-automatically through the Engström Carestation by means of the nitrogen wash-out/wash-in method with a FiO_2_ change of 0.1 [14]. To maintain hemodynamic stability (mean arterial pressure > 60 mmHg) and to avoid instability the animals were treated by continuous central venous noradrenaline infusion or repetitive infusion of a balanced electrolyte solution was used (Sterofundin; B. Braun Melsungen AG, Germany). Therefore, the PiCCO system was used to guide fluid and catecholamine management [15].

### Statistics

All parameters are presented as mean and standard deviation (± SD) or displayed as vertical/graph bars with mean and standard deviation (± SD). The groups were compared by two-way analysis of variance (ANOVA) with a post-hoc Student–Newman–Keuls test. Normality was evaluated using the Shapiro–Wilk test and the F test was performed to evaluate if the variance of two groups are equal. A *p* value lower than 0.05 was accepted as significant. The software package SigmaPlot 12.5 (Systat Software, San Jose, CA, USA) was used for analysation and graphing the plots.

## Results

**Fig. 1:**
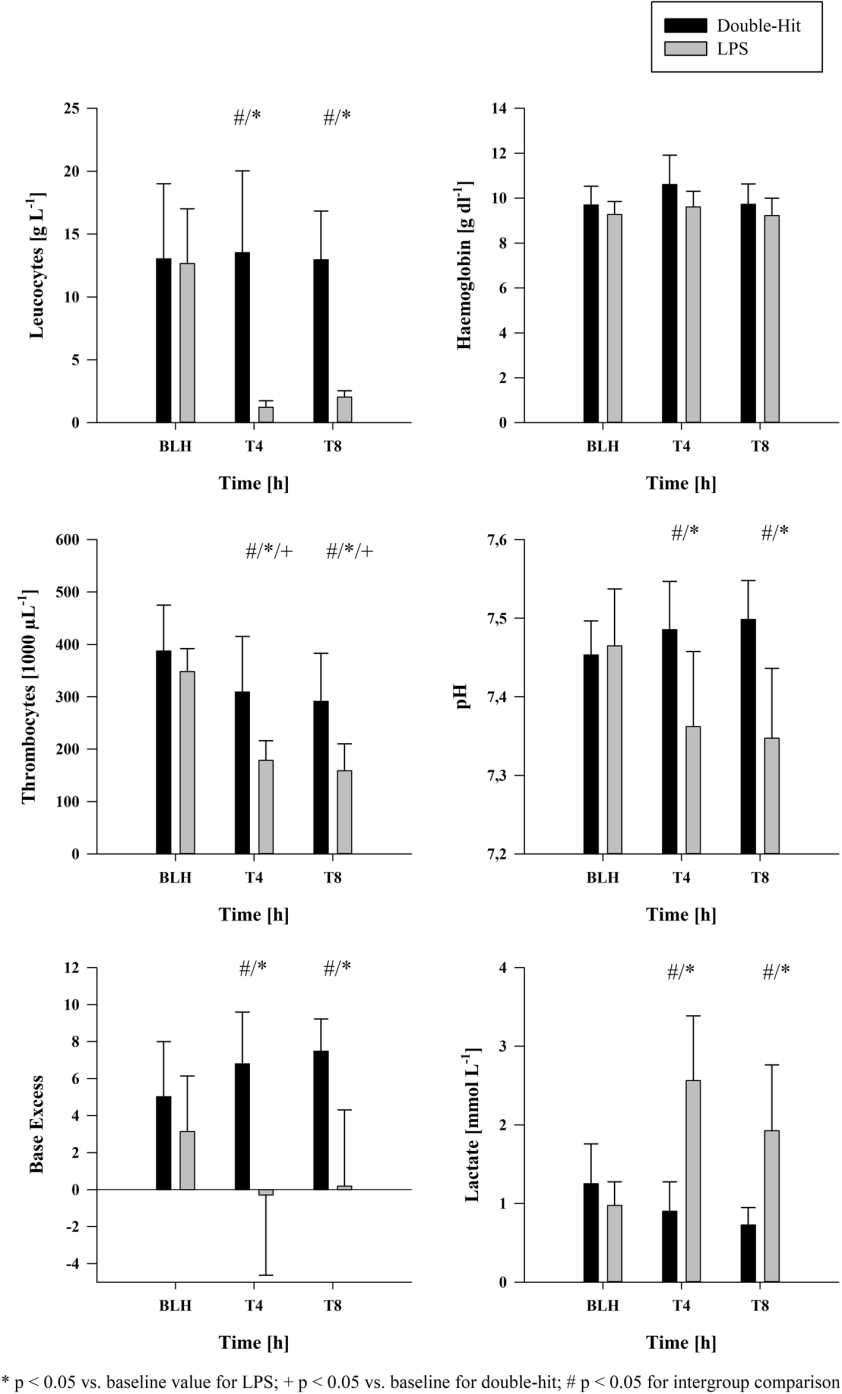
Laboratory parameters

A total of 16 animals were included in this post-hoc analysis (LPS–ARDS: *n* = 8; double-hit ARDS: *n* = 8). Comparable baseline conditions were achieved in terms for all hemodynamic, spirometry and laboratory variables (Tables 1, 2 and Fig. 1).

**Table 1:** Hemodynamic parameters

Parameter	Group	BLH	T0	T4	T8
		Mean (SD)	Mean (SD)	Mean (SD)	Mean (SD)
MAP	Double-Hit	73 (4)	78 (8)#	70 (8)	70 (7)
[mmHg]	LPS	65 (9)	67 (8)	65 (5)	63 (6)
HR	Double-Hit	101 (14)	104 (28)	120 (45)	103 (19)
[min^−1^]	LPS	78 (10)	80 (11)	120 (16)*	144 (12)#/*
mPAP	Double-Hit	20 (3)	33 (2)^#/^*	28 (4)*	23 (4)
[mmHg]	LPS	15 (3)	25 (3)*	35 (3)^#/^*	30 (3)^#/^*
CO	Double-Hit	4.04 (0.61)	3.93 (0.97)	3.22 (0.23)*	3.69 (0.23)
[l min^−1^]	LPS	3.22 (0.54)	3.52 (0.64)	4.86 (1.24)^#/^*	5.58 (1.40)^#/^*
PCWP	Double-Hit	9 (1)	10 (1)	10 (1)	10 (2)
[mmHg]	LPS	8 (2)	10 (1)*	8 (2)	10 (2)*
GEDVI	Double-Hit	501 (92)	493 (114)	427 (74)^#/^*	440 (72)*
[ml m^−2^]	LPS	486 (65)	514 (85)	530 (100)	522 (107)
EVLWI	Double-Hit	11.24 (0.82)	21.27 (5.08)^#/^*	18.02 (2.77)*	16.85 (3.94)*
[ml kg^−1^]	LPS	11.63 (2.28)	12.46 (11.13)	14.89 (1.75)*	15.61 (2.26)*
Norepinephrine	Double-Hit	0 (0)	0.62 (0.79)	0.11 (0.12)	0.11 (0.16)
[mg^−1^]	LPS	0.03 (0.09)	0.01 (0.03)	0.62 (0.55)	1.18 (1.05)^#/^*

^*^Indicates *p* < 0.05 vs. baseline value. # indicates *p* < 0.05 in intergroup comparison

MAP: mean arterial pressure; HR: heart rate; mPAP: mean arterial pulmonary pressure; CO: cardiac output; PCWP: pulmonary capillary wedge pressure; GEDVI: global end-diastolic volume index; EVLWI: end-diastolic lung water index;

**Table 2:** Spirometry parameters

Parameter	Group	BLH	T0	T4	T8
		Mean (SD)	Mean (SD)	Mean (SD)	Mean (SD)
PaO_2_/FiO_2_	Double-Hit	367 (64)	76 (35)^#/^*	225 (54)*	212 (79)*
[mmHg]	LPS	509 (41)	470 (51)	238 (57)*	144 (59)*
FRC	Double-Hit	535 (54)	222 (79)	373 (63)	375 (60)
[ml]	LPS	779 (490)	688 (488)	402 (88)	363 (165)
Ppeak	Double-Hit	16 (0)	28 (4)^#/^*	25 (4)*	24 (4)*
[mbar]	LPS	15 (2)	17 (2)*	26 (5)*	28 (5)*
Pmean	Double-Hit	8 (0)	14 (2)^#/^*	14 (3)*	12 (3)*
[mbar]	LPS	8 (1)	9 (1)	12 (2)*	14 (3)*
PEEP	Double-Hit	4 (0)	5 (1)	8 (3)#/*	7 (2)*
[cm H_2_O]	LPS	4 (0)	5 (0)	6 (2)	8 (2)*
MV	Double-Hit	7.36 (1.15)	7.87 (1.02)^#^	8.85 (1.50)^#/^*	9.11 (1.37)^#/^*
[l min^−1^]	LPS	6.48 (0.58)	6.56 (0.53)	7.55 (0.97)*	7.58 (0.96)*
RR	Double-Hit	37 (4)	38 (4)^#^	45 (5)^#/^*	46 (5)^#/^*
[min^−1^]	LPS	32 (4)	30 (5)	33 (5)	35 (6)
Compliance	Double-Hit	17.08 (1.78)	8.80 (2.16)^#/^*	11.39 (2.79)*	10.88 (2.30)*
[ml/mbar]	LPS	24.94 (4.60)	20.31 (3.69)*	13.15 (2.22)*	12.87 (1.84)*

^*^Indicates *p* < 0.05 vs. baseline value. # indicates *p* < 0.05 in intergroup comparison

PaO_2_: arterial oxygen; FiO_2_: fraction of inspired oxygen; PaO_2_/FiO_2_: oxygen index; FRC: functional residual capacity; MV: minute ventilation; Ppeak: peak inspiratory pressure; Pmean: mean airway pressure; PEEP: positive end-expiratory pressure; RR: respiratory rate

### Hemodynamic

After ARDS induction, a significant increase of the mean arterial pressure (MAP) was measured in the double-hit group compared to the baseline value for T0 (78 ± 8 vs. 73 ± 4; *p* < 0.05; Table 1). Likewise, mean arterial pulmonary pressure (mPAP) increased significantly in both groups over time (33 ± 2/28 ± 4 vs. 20 ± 3; T0/4 vs. BLH for double-hit; 25 ± 3/35 ± 3/30 ± 3 vs. 15 ± 3; T0/4/8 vs. BLH for LPS; both *p* < 0.05; Table 1). Furthermore, a significantly higher mPAP was measured in the double-hit group compared to LPS for T0 (33 ± 2 vs. 25 ± 3; *p* < 0.05, Table 1). This effect is reversed over the experiment. In the LPS group statistically elevated mPAP values were detected 4 and 8 h after ARDS induction compared to the double-hit group (35 ± 3/30 ± 3 vs. 28 ± 4/23 ± 4; T4/T8 LPS vs. double-hit; *p* < 0.05; Table 1). Statistical relevant changes in the heart rate were only observed in the LPS group (120 ± 16/144 ± 12 vs. 78 ± 10; T4/T8 vs. BLH; *p* < 0.05; Table 1). At T8, the heart rate was significantly higher in the LPS group compared to the double-hit group (144 ± 12 vs. 103 ± 19; *p* < 0.05; Table 1). In a similar way the cardiac output (CO) increased over time in the LPS group (4.86 ± 1.24/5.58 ± 1.40 vs. 3.22 ± 0.54; T4/T8 vs. BLH; *p* < 0.05; Table 1). Only at T4 an elevated CO was measured in the double-hit group (3.22 ± 0.23 vs. 4.04 ± 0.61; *p* < 0.05; Table 1). Significant intergroup changes with a higher CO were detected for LPS (4.86 ± 1.24/5.58 ± 1.40 vs. 3.22 ± 0.23/3.69 ± 0.23; T4/T8 LPS vs. double-hit; *p* < 0.05; Table 1). Pulmonary capillary wedge pressure (PCWP) was only significantly elevated in the LPS group at T0 and T4 compared to baseline values (10 ± 1/10 ± 2 vs. 8 ± 2; *p* < 0.05; Table 1). Global end-diastolic volume index (GEDVI) was statistically lower at T4 and T8 in the double-hit group compared to baseline (427 ± 74/440 ± 72 vs. 501 ± 92; *p* < 0.05; Table 1). Further at T4, the GEDVI was significant lower in the double-hit compared to the LPS group (427 ± 74 vs. 530 ± 100; *p* < 0.05; Table 1). End-diastolic lung water index (EVLWI) values were elevated statistically in the double-hit group compared to LPS at T0 (21.27 ± 5.08 vs. 12.46 ± 11.13; *p* < 0.05; Table 1). Additional, EVLWI showed significant elevated values compared to the baseline in both groups (21.27 ± 5.08/18.02 ± 2.77/18.85 ± 3.94 vs. 11.24 ± 0.82; T0/T4/T8 vs. BLH for double-hit; 14.89 ± 1.75/15.61 ± 2.26 vs. 11.63 ± 2.28; T4/T8 vs. BLH for LPS; both *p* < 0.05; Table 1). In the LPS group, statistically higher doses of norepinephrine were used compared to the double-hit group (1.18 ± 1.05 vs. 0.11 ± 0.16; LPS vs. double-hit for T8; *p* < 0.05; Table 1). Furthermore, the norepinephrine dose was elevated compared to baseline conditions in the LPS group at T8 (1.18 ± 1.05 vs. 0.03 ± 0.09; *p* < 0.05; Table 1).

### Respiratory

After ARDS induction, the oxygen index dropped significantly in the double-hit group and remained significantly lower over the time (76 ± 35/225 ± 54/212 ± 79 vs. 367 ± 64; T0/T4/T8 vs. BLH; *p* < 0.05; Table 2). Intergroup differences for the oxygen index were only measured at *T*0 with lower values in the double-hit group (76 ± 35 vs. 470 ± 51; *p* < 0.05; Table 2). Reduced oxygen indices in the LPS group with statistical significance were seen at T4 and T8 compared to baseline (238 ± 57/144 ± 59 vs. 509 ± 41; *p* < 0.05; Table 2). No changes over time for the functional residual capacity in both groups were detected. Higher peak inspiratory pressures (*P*_peak_) after ARDS induction were reported in both groups over the whole experiment compared to baseline (28 ± 4/25 ± 4/24 ± 4 vs. 16 ± 0; T0/T4/T8 vs. BLH for double-hit; 17 ± 2/26 ± 5/28 ± 5 vs. 15 ± 2; T0/T4/T8 vs. BLH for LPS; both *p* < 0.05, Table 2). Furthermore, elevated *P*_peak_ values were measured at T0 in the double-hit group compared to LPS (28 ± 4 vs. 17 ± 2; *p* < 0.05; Table 2). In addition to the *P*_peak_ results, the mean airway pressure (*P*_mean_) was elevated after ARDS induction in both groups (14 ± 2/14 ± 3/12 ± 3 vs. 8 ± 0; T0/T4/T8 vs. BLH for double-hit; 12 ± 2/14 ± 3 vs. 8 ± 1; T4/T8 vs. BLH for LPS; *p* < 0.05, Table 2). At T0, higher *P*_mean_ values were detected in the double-hit group compared to LPS (14 ± 2 vs. 9 ± 1; *p* < 0.05; Table 2). Statistically elevated positive end-expiratory pressure (PEEP) values were measured at T4 and T8 compared to the baseline in the double-hit group (8 ± 3/7 ± 2 vs. 4 ± 0; *p* < 0.05; Table 2). Here, T4 showed significant higher PEEP levels in the double-hit group compared to LPS (8 ± 3 vs. 6 ± 2; *p* < 0.05; Table 2). In the LPS group, PEEP was only elevated at the end of the experiment (8 ± 2 vs. 4 ± 0; T8 vs. BLH; *p* < 0.05; Table 2). The minute ventilation increased significantly in the double-hit group over the experiment (7.87 ± 1.02/8.85 ± 1.50/9.11 ± 1.37 vs. 7.36 ± 1.15; T0/T4/T8 vs. BLH; *p* < 0.05; Table 2). Similar results were seen in the LPS group at T4 and T8 compared to baseline (7.55 ± 0.97/7.58 ± 0.96 vs. 6.48 ± 0.58; *p* < 0.05; Table 2). A statistically elevated minute ventilation between the groups was detected at T0, T4 and T8 with increased values in the double-hit group (7.87 ± 1.02/8.85 ± 1.50/9.11 ± 1.37 vs. 6.56 ± 0.53/7.55 ± 0.97/7.58 ± 0.96; *p* < 0.05; Table 2). The respiratory rate significantly changed in the same way (38 ± 4/45 ± 5/46 ± 5 vs. 30 ± 5/33 ± 5/35 ± 6; T0/T4/T8 double-hit vs. LPS; *p* < 0.05; Table 2). In the double-hit group, the respiratory rate was elevated at T4 and T8 compared to baseline (45 ± 5/46 ± 5 vs. 37 ± 4; *p* < 0.05; Table 2). The compliance decreased immediately after ARDS induction and remained significantly lower over the experiment (8.80 ± 2.16/11.39 ± 2.79/10.88 ± 2.30 vs. 17.80 vs. 1.78; T0/T4/T8 vs. BLH for double-hit; 20.31 ± 3.69/13.15 ± 2.22/12.87 ± 1.84 vs. 24.94 ± 4.60; T0/T4/T8 vs. BLH for LPS; both *p* < 0.05; Table 2). A significantly intergroup difference was detected at T0 with a lower compliance in the double-hit group compared to LPS (8.80 ± 2.16 vs. 20.31 ± 3.69; *p* < 0.05; Table 2). The tissue wet to dry ratio as surrogate of oedema formation did not differ between the groups (double-hit 7.6 ± 1.5 vs. LPS 6.6 ± 1.2; *p* > 0.05).

### Laboratory

**Fig. 2:**
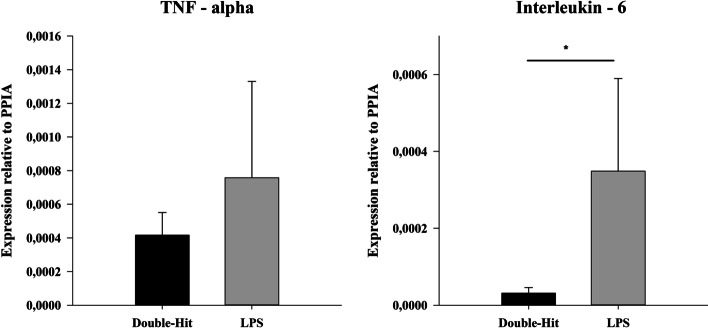
Pulmonary expressions of inflammatory markers

A significant drop of the number of leucocytes was measured in the LPS group at T4 and T8 compared to baseline values (1.23 ± 0.46/2.01 ± 0.42 vs. 12.69 ± 4.02; *p* < 0.05; Fig. 1). In addition, this drop was significantly compared to the double-hit group (1.23 ± 0.46/2.01 ± 0.42 vs. 13.56 ± 5.61/12.98 ± 3.35; T4/T8 LPS vs. double-hit; *p* < 0.05; Fig. 1). Haemoglobin levels remained stable in both groups over time, whereas thrombocytes dropped similar to the leucocytes in the LPS group. Significant inner- and intergroup differences in the number of thrombocytes were measured at T4 and T8 compared to the double-hit group (178 ± 35/158 ± 47 vs. 348 ± 41; T4/T8 vs. BLH for LPS; 178 ± 35/158 ± 47 vs. 309 ± 92/291 ± 79; LPS vs. double-hit; both *p* < 0.05; Fig. 1). Furthermore, thrombocytes remained lower at T4 and T8 in the double-hit group compared to baseline (309 ± 92/291 ± 79 vs. 387 ± 75; *p* < 0.05; Fig. 1). PH, base excess (BE) and lactate levels showed similar alterations (Fig. 1). Significant lower pH and BE levels were seen at T4 and T8 in the LPS group compared to baseline and the double-hit group (7.36 ± 0.08/7.34 ± 0.08 vs. 7.46 ± 0.06; T4/T8 vs. BLH for pH LPS; 7.36 ± 0.08/7.34 ± 0.08 vs. 7.48 ± 0.05/7.49 ± 0.04; T4/T8 LPS vs. double-hit for pH; − 0.23 ± 4.02/0.13 ± 3.87 vs. 3.13 ± 2.86; T4/T8 vs. BLH for BE LPS; − 0.23 ± 4.02/0.13 ± 3.87 vs. 6.85 ± 2.43/7.47 ± 1.55; T4/T8 LPS vs. double-hit for BE; all *p* < 0.05; Fig. 1). Lactate levels were increased in the LPS group compared to the double-hit group (2.56 ± 0.73/2.01 ± 0.73 vs. 0.90 ± 0.32; T4/T8 vs. BLH for LPS; 2.56 ± 0.73/2.01 ± 0.73 vs. 0.91 ± 0.38/0.74 ± 0.24; LPS vs. double-hit for T4/T8; all *p* < 0.05; Fig. 1). The post-mortem pulmonary expression of TNF-alpha showed no differences (1.35e−04 ± 6.73e−05 vs. 7.58e−04 ± 5.73e−04; double-hit vs. LPS; *p* > 0.05; Fig. 2), whereas the expression of interleukin-6 (IL-6) was significantly elevated in the LPS group compared to the double-hit group (2.41e−04 ± 1.08e−04 vs. 1.45e−05 ± 7.26e−06; LPS vs. double-hit; *p* < 0.05; Fig. 2).

## Discussion

In the present post-hoc analysis of two different animal ARDS models, we investigated similarities and/or differences of macroscopic findings to identify a pre-clinical model with the most suitable clinical features seen in relation to human ARDS. Three major findings are:

1. both ARDS models are feasible and reproducible and contribute to an impairment of the gas exchange, whereas
2. the LPS-induced ARDS caused the most severe cardiovascular and metabolic insufficiency and
3. the double-hit model impressed with higher mechanical ventilator settings and altered pulmonary mechanics.

Many different animal models exist to mimic human ARDS and its pathophysiological features to help better understand this syndrome [3]. The chosen animal model should accurately reproduce the various patterns of the disease. Pigs used as a model for lung injury provide numerous advantages compared to rodents: the anatomy, genetics, and physiology are remarkably similar to humans [16]. There is no doubt, when chosen the right model, that acute lung injury models with pigs will offer new crucial findings in the complex pathophysiology of ARDS in the future with innovative renewals from bench to (human) bedside. In the following, the findings of our post-hoc analysis are classified in the context of alterations seen in human ARDS.

### Focus hemodynamic system

Hemodynamic alterations are often seen in ARDS. Up to 60% of the patients experience hemodynamic failure due to (1) pulmonary hypertension and acute cor pulmonale, (2) vasoplegia and (3) arterial remodeling due to sepsis-induced vascular dysfunction [17, 18]. To improve perfusion, cardiac output and to measure EVLWI and balance the infusion therapy, it is recommended to use transpulmonary thermodilution systems as done in our studies [19]. The hemodynamic changes mentioned were observed more frequently in the LPS group in the presented analysis. Heart rate, mean arterial pulmonary pressure, cardiac output and wedge pressure were elevated similar to human septic ARDS. Mean arterial blood pressure was kept stable in both groups. However, this only happened due to significantly higher norepinephrine doses in the LPS group. This observation may suggest a septic-induced vascular dysfunction in these animals. The role of different catecholamines in (bovine) ARDS has not been investigated so far. Thereby, the LPS induced ARDS model could offer a suitable option. Furthermore, EVLWI was elevated in both groups. One key finding in the pathophysiology of ARDS is the development of lung oedema. Elevated EVLWI reflects the persistence of pulmonary edema. Similar results were found in a septic model in minipigs. In this study, EVLWI was elevated over time after fecal peritonitis [20]. As seen here, both models show a significant damage to the alveolar capillary unit which causes the characteristic oedema. The measurement of the PCWP, as a surrogate marker for a cardiogenic pulmonary oedema, supports these findings. Neither the ARDS induced with LPS nor the double-hit ARDS lead to relevant changes in the PCWP values. It is well-known, that elevated PCWP values are associated with poor outcome in ARDS due to right ventricular failure [21, 22]. In our study, it remains unclear why the PCWP do not raise in the double-hit model. It is reported that especially oleic acid infusion elevates significantly the PCWP in an ARDS model in dogs [23]. To summarize, different fluid, catecholamines and transfusion therapy regimes could be addressed and studied with both models to reduce the impact of an edema and ameliorate the hemodynamics in ARDS.

### Focus respiratory system

In the past, the adverse effects of mechanical ventilation in patients with ARDS are discussed [24]. The inhomogeneity of gasless regions up to hyperinflated areas are present in the lung and contribute to the ventilator-induced lung injury (VILI) [25]. Furthermore, lung oedema, anatomic variations and the reduced ventilatable lung tissue make “non-adverse” ventilation difficult. Not higher tidal volumes itself damage the lung, it is the mechanical power delivered by the mechanical ventilation that affects the lung and the development of VILI [25]. Respiratory rate, mean and peak airway pressures as well as PEEP are other main determinants of mechanical power. Optimal PEEP in patients is still discussed. For example, some authors recommend higher PEEP in patients with the highest recruitable lung parenchyma and the most hypoxemic patients [26]. As seen in our analysis, higher peak and mean airway pressures were generated directly after ARDS induction and maintained over time in the double-hit model. Furthermore, respiratory rate and minute ventilation was increased in the double-hit model compared to LPS. The impact of different animal models to mechanical ventilation parameters has not been investigated so far, whereas different types of ventilation modes and their impact of VILI and inflammation were well-analyzed. In an ARDS model with piglets, the influence of spontaneous breathing or mechanical ventilation on abdominal oedema and inflammation was investigated [27]. Decreased lung compliance is another pathophysiological finding in human ARDS. Since COVID-19, it is known that different compliance phenotypes in human ARDS exist and the research focus on this parameter became more popular [28]. In both groups, the compliance decreased immediately after ARDS induction and remained lower over time. This effect was more worsen in the double-hit group. Yet, changes in the lung compliance have been investigated in pigs during pronation in ARDS [27]. Due to the reduced compliance, hypoxemia was more severe in the double-hit model immediately after ARDS induction. The PEEP levels also increased more in this group. Determining the optimal PEEP settings in clinical routines is challenging, especially when protective ventilation strategies must be followed. Optimizing PEEP research is often combined with the use of the EIT. In a bovine double-hit ARDS model, using lavage and high tidal volumes to induce lung injury, similar results were found as reported in our study [29]. In conclusion, the double-hit model offers advantages in respiratory research when focusing on different mechanical ventilator strategies (a.e. influence of different PEEP and airway pressures on lung inflammation).

### Focus inflammation

LPS-induced and sepsis-associated ARDS is characterized by systemic inflammatory changes: imbalance of inflammatory response, immune dysfunction and mitochondrial damage [30]. The pathology of sepsis-induced ARDS is extremely complex. IL-6 plays a key role in promoting pulmonary vascular dysfunction, microthrombi and failure of hypoxic pulmonary vasoconstriction (HPV) with in consequence elevated pulmonal arterial pressures [31]. In our study, elevated expression of IL-6 was observed in the lung tissue from LPS-animals probably contributing to significant higher mPAP overtime in this group. Furthermore, elevated lactate levels, decreased base excess and lower pH values may reflect tissue hypoxia and changes in lactate metabolism [32]. Especially elevated lactate levels over time are associated with higher mortality in critical ill patients [32]. In addition, the drops in the white blood cell count and thrombocytes are also common in sepsis-induced ARDS. These “sepsis-like changes” were seen only in the LPS group in our comparison. Gram-negative and LPS-associated sepsis is one of the most causes in human ARDS and has clinical relevance [33]. Nevertheless, a major disadvantage of this model is that the response to endotoxin has significant interspecies variation, with dogs being more tolerant to endotoxin exposure than pigs, sheep or humans [33]. In conclusion, in the double-hit model no remarkable inflammatory changes were observed. The LPS-induced ARDS model offers clear advantages when focusing on studies with inflammatory changes.

Our study has some limitations. (1) The short follow-up period of 8 h addresses only the acute phase of an ARDS. Long-term effects will not be shown. (2) To reduce confounding variables all pigs were of the same gender, a situation not seen in clinical daily praxis. (3) Concerning the double-hit model, it remains unclear in which part the oleic acid infusion or the bronchoalveolar lavage cause the observed lung injury. (4) Despite all the similarities between humans and pigs, results of animal studies need to be translated to clinical practice.

In conclusion, the LPS-induced ARDS caused the most severe cardiovascular and metabolic insufficiency and has clinical relevance due the gram-negative nature of LPS. The double-hit model impressed with higher mechanical ventilator settings. The results are in conclusion with findings in sheep and humans that support the value of different animal models [34]. We can conclude that the different causes of ARDS resulted in the same clinical starting point with severe gas exchange problems. However, in the short time of 8 h in our experiments the underlying causes of ARDS affected the clinical properties of this models in the further course. The choice of which ARDS animal model to use must be carefully considered based upon the focus of the study. Acid aspiration, hyperoxia and bleomycin models also exist and needed to be addressed in further comparison studies to identify an ARDS animal model with the most clinical features and accordance of ARDS in the future.

## Data Availability

The data sets used and/or analysed during the current study are available from the corresponding author on reasonable request.
